# The fastest animals and vehicles are neither the biggest nor the fastest over lifetime

**DOI:** 10.1038/s41598-018-30303-1

**Published:** 2018-08-27

**Authors:** A. Bejan, U. Gunes, J. D. Charles, B. Sahin

**Affiliations:** 1 Duke University, Department of Mechanical Engineering and Materials Science, Durham, NC 27708-0300 USA; 2 Blue Origin, 21218 76th Ave S, Kent, WA 98032 USA; 3 Yildiz Technical University, Department of Naval Architecture and Marine Engineering, Besiktas, Istanbul, 34349 Turkey

## Abstract

Here we show how the size of a body affects its maximum average speed of movement through its environment. The theoretical challenge was to predict that ‘outliers’ must exist, such as the cheetah for terrestrial animals and the jet fighter for airplanes. We show that during a travel that starts from rest and continues at cruising speed, the body size for minimum travel time, or maximum average speed, is not the biggest. The results are compared with extensive data for military aircraft for chase, attack and reconnaissance, in addition to data for commercial aircraft. The paper also explains why in earlier studies of flying (animals, airplanes) the airplane data deviated upward (toward greater speeds) relative to the theoretical trend followed by flying animals, and why the fastest animal flyers are one thousand times smaller than the fastest swimmers. Unlike the biggest animals and airplanes (elephant, whale, commercial jet), which move constantly, the fastest animals and airplanes spend most of their lives at rest. When judged for speed averaged over lifetime, the fastest ‘sprinters’ are in fact the slowest movers (as in Aesop’s fable ‘The Tortoise and the Hare’).

## Introduction

**Figure 1:**
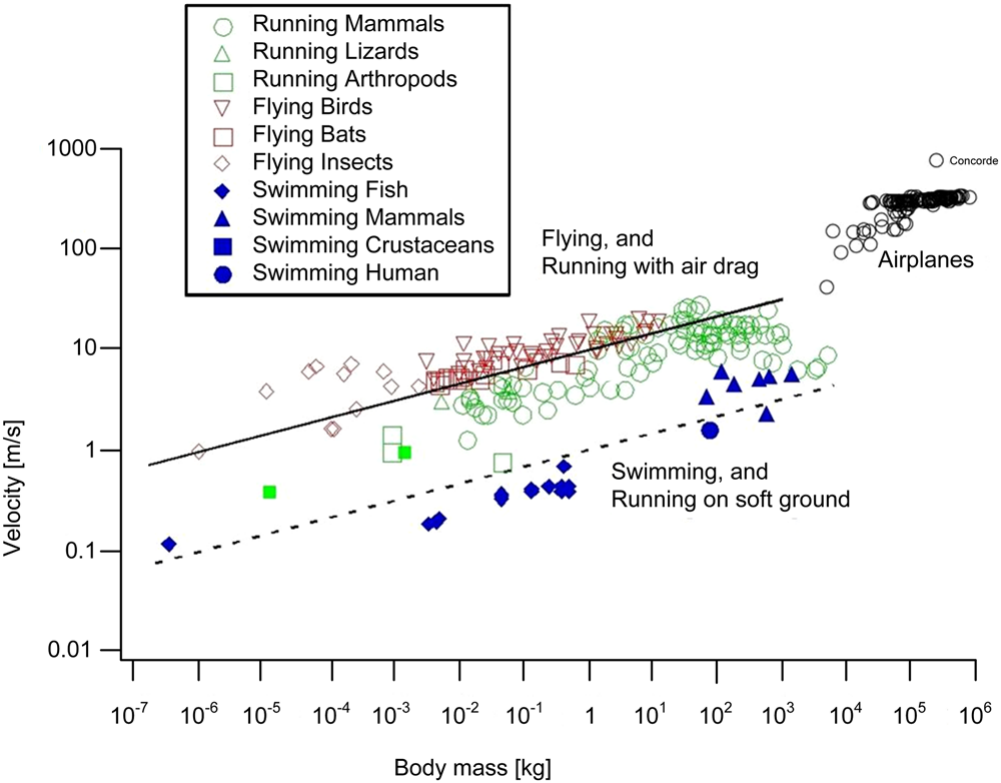
Animal speed-mass data (fliers, runners, swimmers) compiled from sources indicated in ref.^1^.

Bigger bodies tend to move faster on land, in water, and in the air. This broad trend is supported by the speed-size data collected from many sources for flying, running and swimming (Fig. 1)^1^. On this unifying background, outliers exist. The burst speed of the cheetah is greater than the steady speed of the elephant. The tuna can swim faster than the steady whale. There are outliers that are even more distant from the general trend, but they should not be confused with the fastest runners, swimmers and fliers. For example, there are insects (e.g., the flea) that snap and jump at speeds much greater than what their speed-mass scaling law would predict. The ant *odontomachus* holds this kind of speed record (of order 200 km/h) by snapping its jaws shut, in order to jump to the side, to get out of the way of danger. This is a one-shot event, not cyclical locomotion. Most of her life, this ant walks on land at ant speed, in accord with the speed-mass scaling law for animal locomotion^1^.

**Figure 2:**
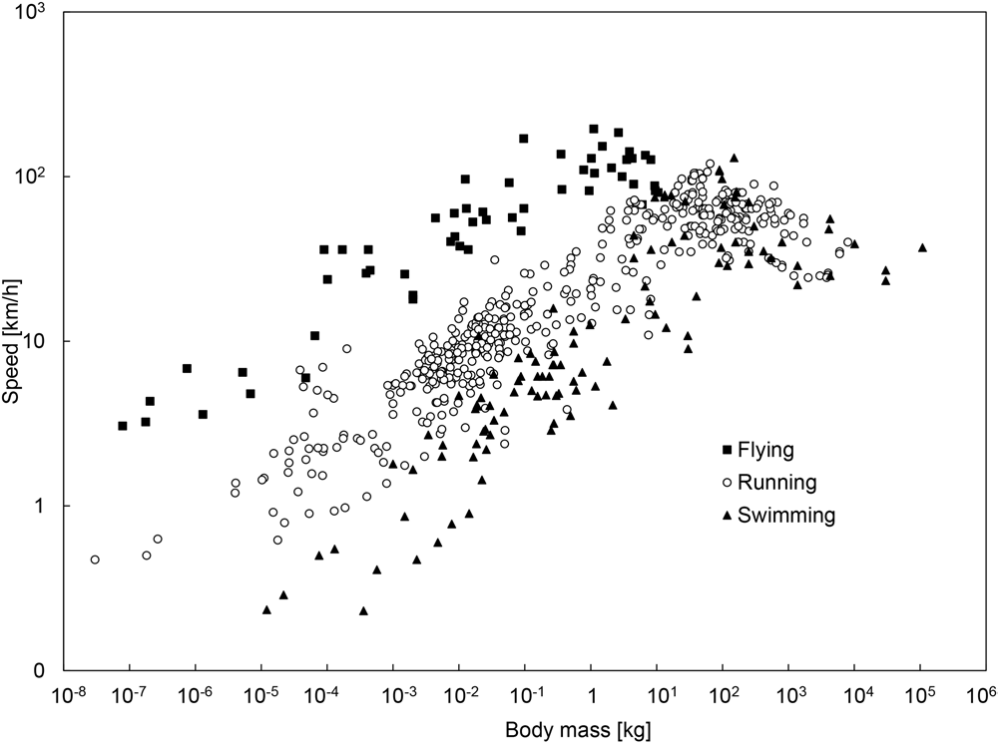
Animal speed-mass data compiled after Hirt *et al*.^2^, showing that the peak speed belongs to animals that are close to but not the biggest. The data are compiled from sources indicated in ref.^2^.

Hirt *et al*.^2^ addressed this aspect of animal locomotion with a model chosen to account for the starting (accelerating) period in the movement of the fastest animals. The model consisting of elastic fast-twitch fibers was constructed such that the speed emerges as a concave function of body size, with its peak at a body size that is not the largest size (Fig. 2). Bejan^3^ observed that the existence of body size for peak speed also rules the evolutionary design of jet fighter aircraft, which is the human made counterpart of the animal of prey, with a high burst speed followed by a long period of inactivity on the ground.

The following analysis is a theoretical treatment of this physics aspect of locomotion. It is a generalization of the unifying theory of animate, inanimate and vehicle movement, which includes the lifetime and life travel of animate and inanimate movement^4^.

Additionally, the following theory accounts for several features of the alignment of locomotion data that have not been questioned. One is the fact that the data for airplanes in Fig. 1 are not aligned with the data for animal fliers. Why are the airplane data trending above the animal speed-mass correlation? Why do the airplane data fall on a steeper line than the data for animal fliers? Moving over to Fig. 2, why does the body size for peak speed decrease in the direction from swimming to running and flying?

## Theory

Consider the body of a vehicle or animal of size M [kg] moving horizontally on the earth’s surface to a distance L. The body starts from rest (V = 0), is then accelerated to the cruising speed V_c_ over the distance L_a_ and time interval t_a_. The body continues its travel at cruising speed V_c_ over the distance L_c_ and time interval t_c_. We ask what size (M) enables the body to travel the distance L = L_a_ + L_c_ the fastest.

The body M moves because it is driven by power. The power comes from an engine (animal, or human made). The power produced by the engine for the purpose of forcing the motion is^4^

1 $$\dot{{\rm{W}}}={\rm{\eta }}\dot{{\rm{Q}}}$$

where $\dot{{\rm{Q}}}=\dot{{\rm{m}}}H$ is the rate of heat transfer that drives the engine, $\dot{{\rm{m}}}\,[{\rm{kg}}/{\rm{s}}]$ is the rate of fuel (or food) consumption, and H [J/kg] is the heating value of the consumed fuel.

**Figure 3:**
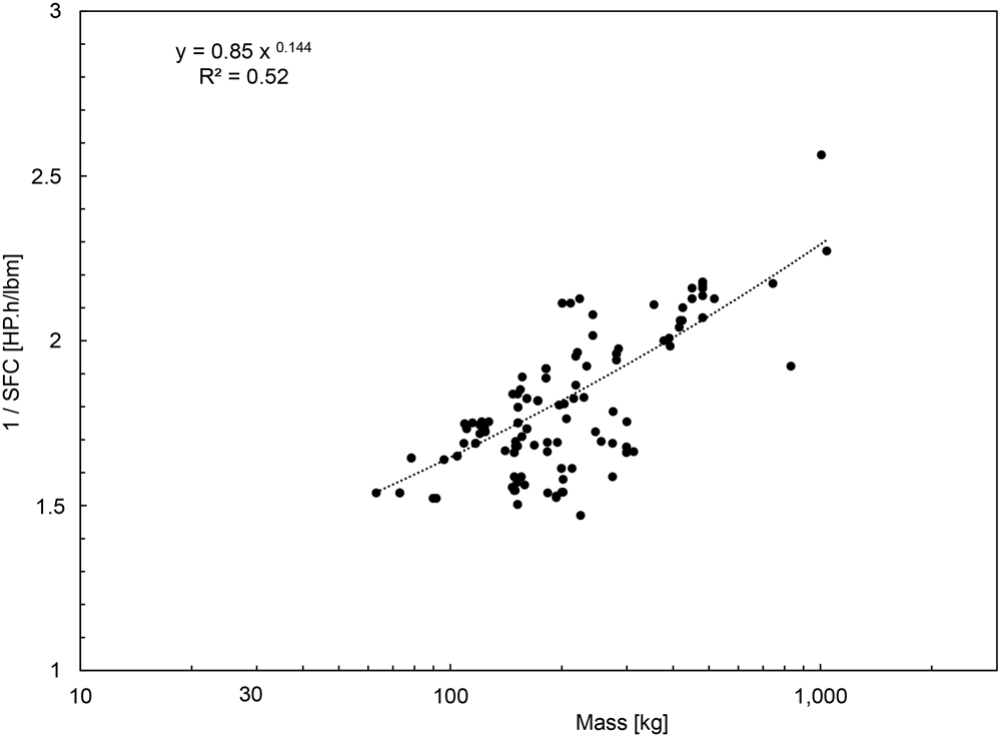
The effect of size on the efficiency of jet engines for aircraft. The data are tabulated in Supplementary Material.

Recent physics articles^5,6^ showed that for maximum efficiency the engine size must have the same scale as the body size M, which is why in the following analysis M is the scale of the body plus the engine. In other words, the moving body has a single size scale, M. Next, the phenomenon of economies of scale requires the efficiency to increase monotonically with the body size,

2 $${\rm{\eta }}={{\rm{C}}}_{{\rm{1}}}{{\rm{M}}}^{{\rm{\alpha }}}$$

where the exponent α is less than 1, for example, α = 1/4 for helicopter engines^7^, and α = 5/12 for animal locomotion (flying, running, swimming)^4^. In Fig. 3 we show that economies of scale are also present in the evolution of jet engines for aircraft, and that the exponent α is of order 0.14. The data^8^ plotted in Fig. 3 are tabulated in Supplementary Material. The R^2^ value is 0.52 and the P value is below 0.001, therefore the correlation is statistically significant^9^. Combining Eqs (1) and (2) we find that the power that drives the body is

3 $$\dot{{\rm{W}}}={{\rm{C}}}_{1}{{\rm{HM}}}^{\alpha }\dot{{\rm{m}}}$$

The power is dissipated in three ways:

(i) To accelerate the body,
(ii) To lift the body (or an equivalent body of water in the case of swimmers^1^), and
(iii) To overcome the drag posed by the medium relative to which the body is moving.

We distinguish two sequential regimes of movement: acceleration, defined such that (ii) and (iii) are negligible compared with (i), and cruise, in which (i) is negligible compared with (ii) and (iii).

## Acceleration

During acceleration, the power exerted by the motor on the body is matched by the force of accelerating the mass (MdV/dt) times the instantaneous speed V,

4 $${{\rm{C}}}_{1}{{\rm{HM}}}^{{\rm{\alpha }}}\dot{{\rm{m}}}={\rm{M}}\frac{{\rm{dV}}}{{\rm{dt}}}{\rm{V}}$$

Integrating this equation from t = 0 to t = t_a_, where V = V_c_, we find

5 $${{\rm{m}}}_{{\rm{a}}}=\frac{{{\rm{M}}}^{1-{\rm{\alpha }}}{{\rm{V}}}_{{\rm{c}}}^{2}}{2{{\rm{C}}}_{1}{\rm{H}}}$$

6 $${{\rm{t}}}_{{\rm{a}}}=\frac{{{\rm{M}}}^{1-{\rm{\alpha }}}{{\rm{V}}}_{{\rm{c}}}^{2}}{2{{\rm{C}}}_{1}{\rm{H}}\overline{{\dot{{\rm{m}}}}_{{\rm{a}}}}}$$

7 $${{\rm{L}}}_{{\rm{a}}}=\frac{{{\rm{M}}}^{1-{\rm{\alpha }}}{{\rm{V}}}_{c}^{3}}{3{{\rm{C}}}_{1}{\rm{H}}\overline{{\dot{{\rm{m}}}}_{{\rm{a}}}}}$$

where m_a_ is the amount of fuel used over the acceleration period, and $\overline{{\dot{{\rm{m}}}}_{{\rm{a}}}}$ is the average fuel consumption rate (m_a_/t_a_) during that period.

The theoretical cruising speed V_c_ emerged^1^ as the trade off between the power spent on lifting (ii) and the power spent on overcoming drag (iii). The cruising speed depends monotonically on body size,

8 $${{\rm{V}}}_{{\rm{c}}}={{\rm{C}}}_{2}{{\rm{M}}}^{1/6}$$

where C_2_ = rg^1/2^ ρ^−1/6^, where ρ is the body density, and r accounts for the medium in which the body is moving: r = (ρ/ρ_a_)^1/3^, where ρ is the body density and ρ_a_ is the density of the ambient. Representative ranges of values are r ~ 10 for fliers, r ~ 1 for swimmers, and 1 < r < 10 for terrestrial locomotion. Combined, Eqs (6) and (8) confirm one side of the trade off noted at the start: bigger bodies take longer to be accelerated to their cruising speed:

9 $${{\rm{t}}}_{{\rm{a}}}=\frac{{{\rm{M}}}^{4/3-{\rm{\alpha }}}{{\rm{C}}}_{2}^{2}}{2{{\rm{C}}}_{1}{\rm{H}}\overline{{\dot{{\rm{m}}}}_{{\rm{a}}}}}$$

## Cruise

The corresponding analysis for the cruising period begins with Eq. (8), where V_c_ = L_c_/t_c_. The engine work spent over L_c_ is

10 $${{\rm{W}}}_{{\rm{c}}} \sim {\rm{r}}{}^{-{\rm{1}}}\,{\rm{Mg}}\,{{\rm{L}}}_{{\rm{c}}}={{\rm{C}}}_{{\rm{1}}}{{\rm{HM}}}^{{\rm{\alpha }}}\overline{{\dot{{\rm{m}}}}_{{\rm{c}}}}{{\rm{t}}}_{{\rm{c}}}$$

The travel time t_c_ is shorter when the body is bigger, and this confirms the second side of the trade off,

11 $${{\rm{t}}}_{{\rm{c}}}=\frac{{{\rm{L}}}_{{\rm{c}}}}{{{\rm{C}}}_{{\rm{2}}}{{\rm{M}}}^{1/6}}$$

## The size trade off

A first glimpse at the trade off that determines the body size for shortest travel time is possible if we assume that the fuel consumption rate is represented by a known constant, ${\dot{{\rm{m}}}}_{{\rm{a}}}={\dot{{\rm{m}}}}_{{\rm{c}}}$. The analysis begins with Eq. (11) by replacing L_c_ with (L − L_a_), where L is fixed and L_a_ is given by Eq. (7). The total travel time, or the inverse of the speed averaged over the distance L, is

12 $$\frac{{{\rm{t}}}_{{\rm{a}}}+{{\rm{t}}}_{{\rm{c}}}}{{\rm{L}}}=\frac{{{\rm{C}}}_{{\rm{2}}}^{{\rm{2}}}}{{{\rm{6C}}}_{{\rm{1}}}{\rm{HL}}\overline{{\dot{{\rm{m}}}}_{{\rm{a}}}}}{{\rm{M}}}^{{\rm{4}}/{\rm{3}}-{\rm{\alpha }}}+\frac{{\rm{1}}}{{{\rm{C}}}_{{\rm{2}}}}{{\rm{M}}}^{-1/6}$$

with the condition that L_a_ < L. Equation (12) can be nondimensionalized by introducing the cruising speed of a reference body of fixed unit mass, M_0_ = 1 kg, namely ${{\rm{V}}}_{0}={{\rm{C}}}_{2}{{\rm{M}}}_{0}^{1/6}$. Equation (12) becomes

13 $$\frac{1}{{\tilde{{\rm{V}}}}_{{\rm{avg}}}}={{\rm{A}}\tilde{{\rm{M}}}}^{4/3-\alpha }+{\tilde{{\rm{M}}}}^{-1/6}$$

where $${\tilde{{\rm{V}}}}_{{\rm{avg}}}={{\rm{V}}}_{{\rm{avg}}}/{{\rm{V}}}_{0}\,{\rm{and}}\,{{\rm{V}}}_{{\rm{avg}}}={\rm{L}}/({{\rm{t}}}_{{\rm{a}}}+{{\rm{t}}}_{{\rm{c}}}),$$

14 $$\frac{1}{{\tilde{V}}_{{\rm{avg}}}}=\frac{{{\rm{t}}}_{{\rm{a}}}+{{\rm{t}}}_{{\rm{c}}}}{{\rm{L}}}\cdot {{\rm{C}}}_{2}{{\rm{M}}}_{0}^{1/6}$$

15 $$\tilde{{\rm{M}}}=\frac{{\rm{M}}}{{{\rm{M}}}_{0}}$$

16 $${\rm{A}}=\frac{{{\rm{C}}}_{2}^{3}{{\rm{M}}}_{0}^{3/2\,-\alpha }}{6{{\rm{C}}}_{1}{\rm{HL}}\overline{{\dot{{\rm{m}}}}_{{\rm{a}}}}}$$

Equation (13) shows that the total travel time has a minimum with respect to body size. Alternatively, the speed averaged over the total travel has a maximum with respect to body size. The design for peak velocity is represented by

17 $${\tilde{{\rm{M}}}}_{{\rm{peak}}}={{\rm{A}}}^{\tfrac{-1}{3/2-\alpha }}\,\,{{\rm{B}}}_{{\rm{M}}}(\alpha )$$

18 $${\tilde{{\rm{V}}}}_{{\rm{peak}}}={{\rm{A}}}^{\tfrac{-1/6}{3/2-\alpha }}\,\,{{\rm{B}}}_{{\rm{V}}}(\alpha )$$

where

19 $${{\rm{B}}}_{{\rm{M}}}(\alpha )={[6(\frac{4}{3}-\alpha )]}^{\frac{-1}{3/2-\alpha }}$$

20 $${{\rm{B}}}_{{\rm{V}}}(\alpha )={({{\rm{B}}}_{{\rm{M}}}^{4/3-\alpha }+{{\rm{B}}}_{{\rm{M}}}^{\frac{-1/6}{3/2-\alpha }})}^{-1}$$

**Figure 4:**
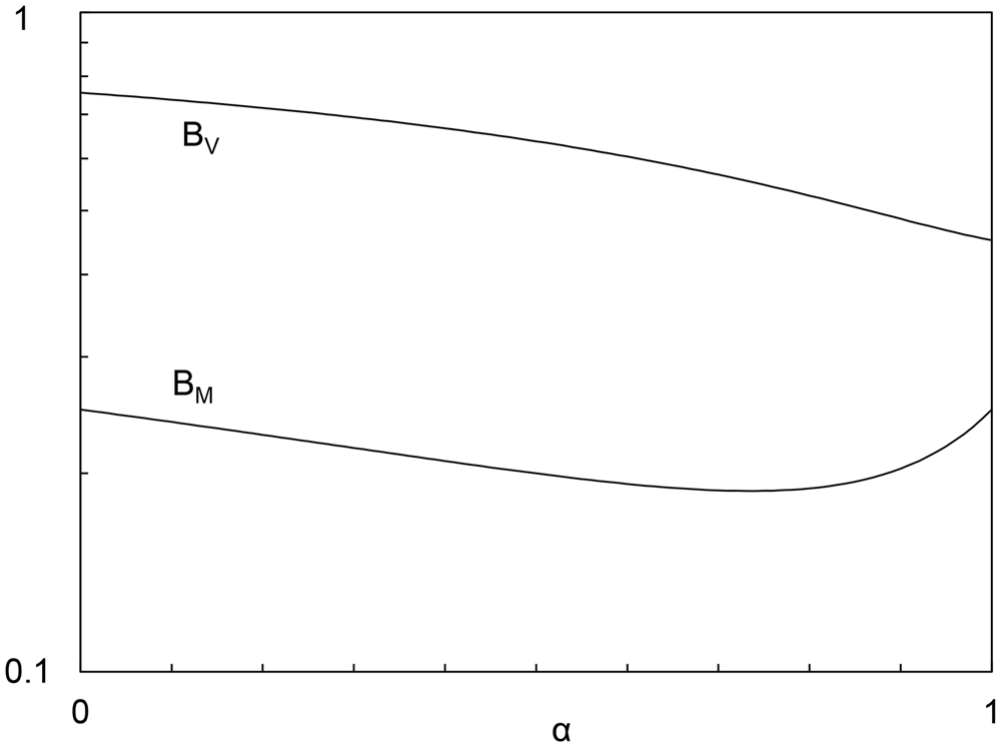
The insensitivity of factors B_M_ and B_v_ to changes in α.

Figure 4 shows that the factors B_M_ and B_V_ are both of order 1, and are relatively insensitive to changes in the exponent α.

These results are in accord with the model of Hirt *et al*.^2^: the largest animal is not necessarily the fastest. The fastest travel emerges at an intermediate size in animals (and vehicles) where the accelerating period is not negligible in comparison with the total travel time. This feature belongs to animals of prey and jet fighters. Another conclusion is that even the fastest [Eqs (17) and (18)] obey the theoretical^1^ proportionality between speed and mass raised to the power 1/6, as in Eq. (8), namely

21 $${\tilde{{\rm{V}}}}_{{\rm{peak}}}=\frac{{{\rm{B}}}_{{\rm{V}}}}{{{\rm{B}}}_{{\rm{M}}}^{1/6}}\,\,{\tilde{{\rm{M}}}}_{{\rm{peak}}}^{1/6}$$

The analysis presented above is based on the simplifying assumption that the fuel consumption rate $\dot{{\rm{m}}}$ is a constant. If we also account for the body size effect on $\dot{{\rm{m}}}$, we can repeat the analysis by replacing $\dot{{\rm{m}}}$ with C_3_M^β^, where, as we show next, the exponent β is approximately 1/2. The β exponent has been found previously in two ways, empirically for commercial airplanes^6^, and theoretically for animals^4^. Specifically, for airplanes the fuel load is roughly M/3, the range is statistically proportional to M^0.64^, and if we take the average speed to be proportional to M^1/6^ we conclude that β ≅ 0.53. For animals, the analysis detailed in ref.^10^ concluded with β = 0.5. This means that in the results presented above C_1_ is replaced by C_1_C_3_, and α is replaced by α + β. In other words, the value of the exponent ${\rm{\alpha }}\lesssim {\rm{0.5}}$, which was used until now, is replaced by an exponent with a value ${\rm{\alpha }}+{\rm{\beta }}\lesssim {\rm{1}}$. Looking at Fig. 4, we see that the change from 0.5 to 1 on the abscissa does not have a meaningful effect on B_M_, B_V_ and Eq. (21).

## The fastest airplanes

**Figure 5:**
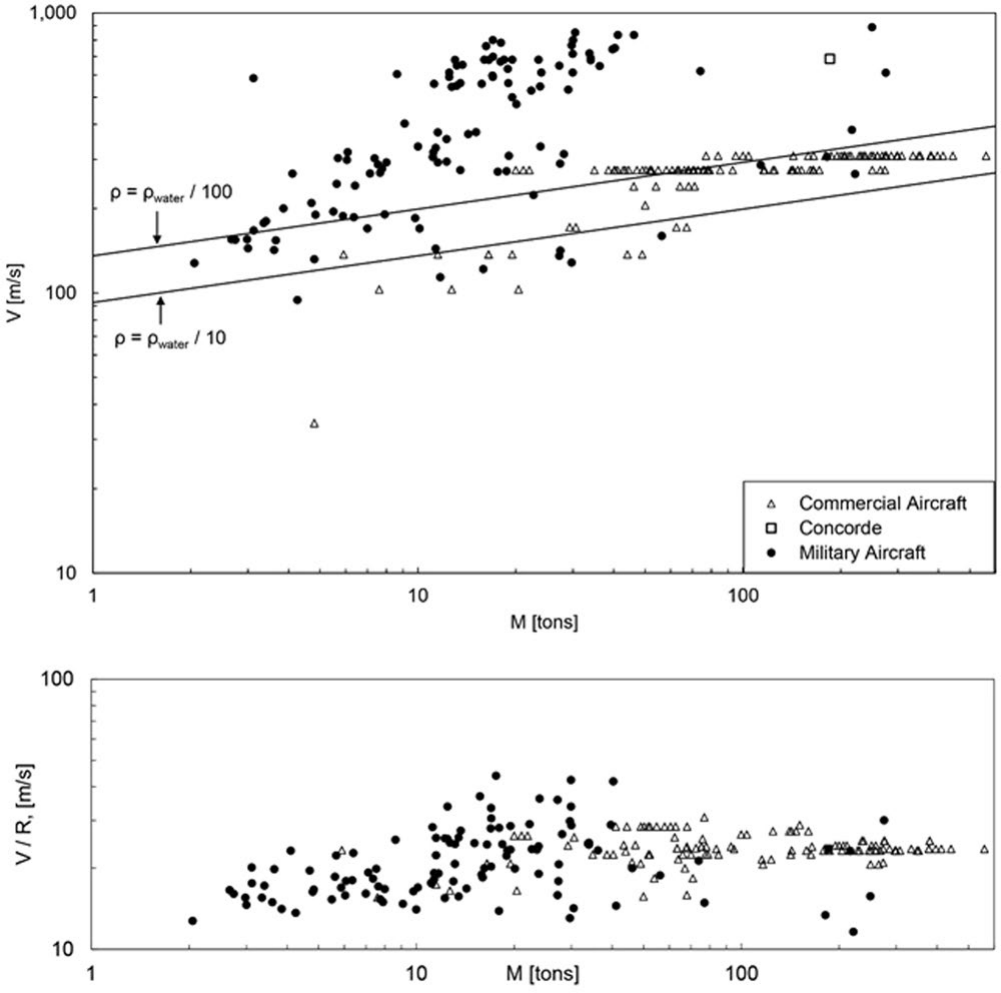
Speed versus size in military and commercial aircraft. Bottom: the military aircraft data collapse on the commercial aircraft data and the predicted theoretical trend when the effect of altitude is included.

**Figure 6:**
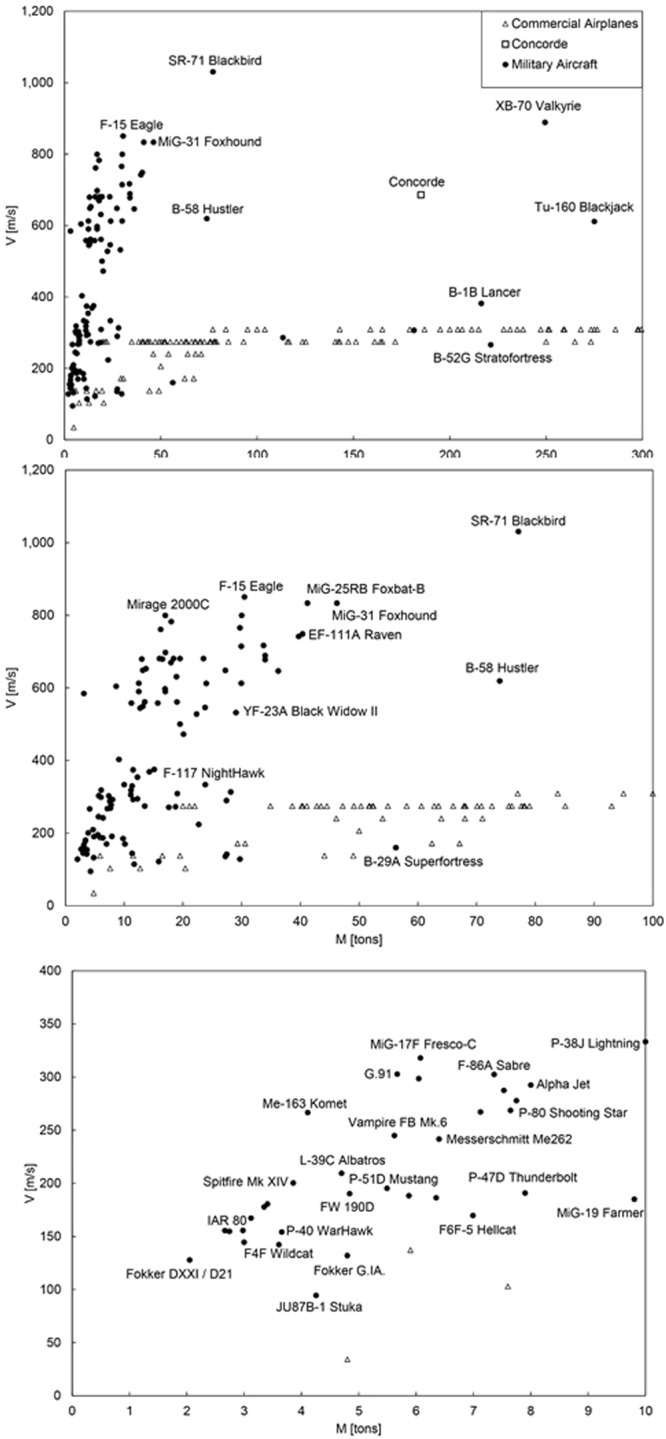
The evolution of speed in the design of military attack aircraft. The data are tabulated in Supplementary Material.

**Figure 7:**
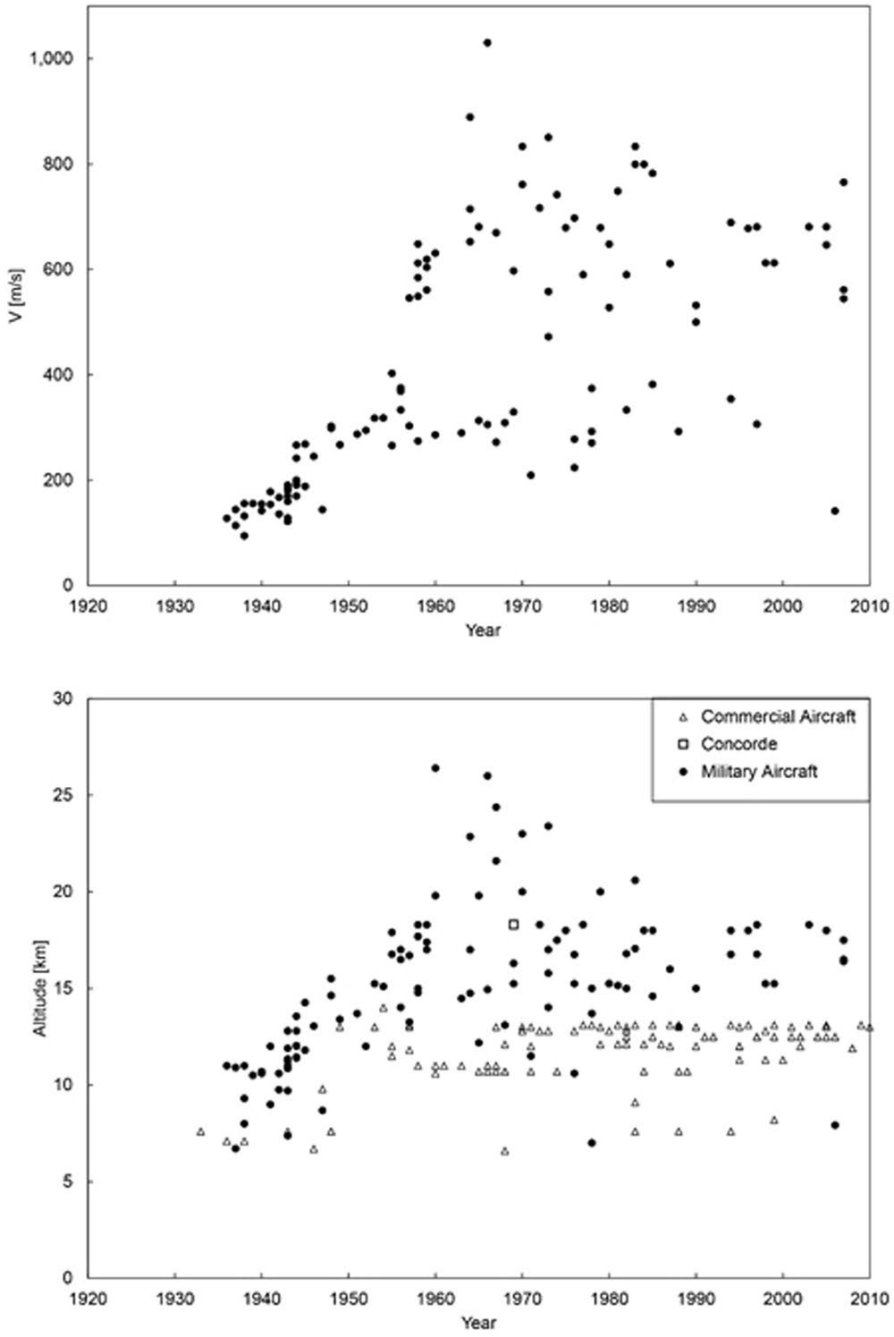
The timeline of the speed and altitude ceiling reached by military aircraft.

The quest for speed in human flight has a rich history dominated by the development of military aircraft for attack and reconnaissance^10^. Highlights are collected chronologically in Fig. 5 (top) and Fig. 6. The data plotted in these figures are tabulated in Supplementary Material. The sequence of frames in Fig. 6 focuses progressively on earlier stages of the development of fast models. Figure 7 (top) shows the evolution of the speeds of military aircraft over time.

The broad view conveyed by Figs 5–7 is that during the past eight decades the fastest models have been joined by even faster models that are also bigger. The theoretical trend derived in Eq. (21) matches the cloud of data of commercial aircraft and early military models compiled in Fig. 5 (top). Several approximating assumptions were made in tracing the two lines, namely r ≈ 10, B_v_ ≈ 1.5 and B_M_ ≈ 0.2. For the average density of the aircraft [ρ in the C_2_ formula under Eq. (8)] we used the method of ref.^11^, which shows that the density of the fuselage of the B747-400 is 31.9 kg m^−3^, or 3 percent of the density of water. We indicated this order of magnitude as the band between ρ = ρ_water_/100 and ρ = ρ_water_/10.

Most of the data for the fastest military models fall above the trend traced with Eq. (21) in Fig. 5 (top). The explanation has its origin in the fact that the early military models flew at subsonic speeds and at altitudes comparable with those of contemporary commercial models. Although the fastest military models since the 1950s have supersonic speeds, they also fly at higher altitudes.

Figure 7 (bottom) complements Fig. 5 (top) by showing the altitude ceiling of each of the models. At higher altitudes the air density is lower, and the r factor (in C_2_, and later in V_peak_) is greater. Specifically, if in the r definition

22 $${\rm{r}}={(\frac{{\rm{\rho }}}{{{\rm{\rho }}}_{{\rm{a}}}})}^{1/3}$$

ρ_a_ is the air density at low altitudes that are comparable with ground level, then the correct r factor that belongs in the theoretical result for V_peak_ [Eq. (21)] is

23 $${\rm{r}}={(\frac{{\rm{\rho }}}{{{\rm{\rho }}}_{{\rm{a}},{\rm{ceiling}}}})}^{1/3}={(\frac{{\rm{\rho }}}{{{\rm{\rho }}}_{{\rm{a}},{\rm{ground}}}})}^{1/3}{\rm{R}}$$

where

24 $${\rm{R}}={(\frac{{{\rm{\rho }}}_{{\rm{a}},{\rm{ground}}}}{{{\rm{\rho }}}_{{\rm{a}},{\rm{ceiling}}}})}^{1/3}$$

The new factor R is monotonically greater than 1 as the ceiling altitude plotted in Fig. 7 (bottom) increases.

## Why airplanes deviate from flying animals

The flying altitude has a predictable effect on the maximum theoretical speed, Eq. (23), because V_peak_ is proportional to r. To bring this effect into view, in Fig. 5 (bottom) we repeated the data of Fig. 5 (top) by plotting on the ordinate V/R in place of V. This way we removed the effect of altitude, in other words, we referenced all the theoretical speeds to the r value that corresponds to air density at ground level.

The R values employed in Fig. 5 (bottom) come from Eq. (24) and the relationship between altitude and atmospheric air density^12^. For example, at an altitude of 10 km the air density is 0.41 kg/m^3^ at −50 °C, while at ground level at 20 °C it is 1.2 kg/m^3^. In this numerical example R is approximately 0.15.

**Figure 8:**
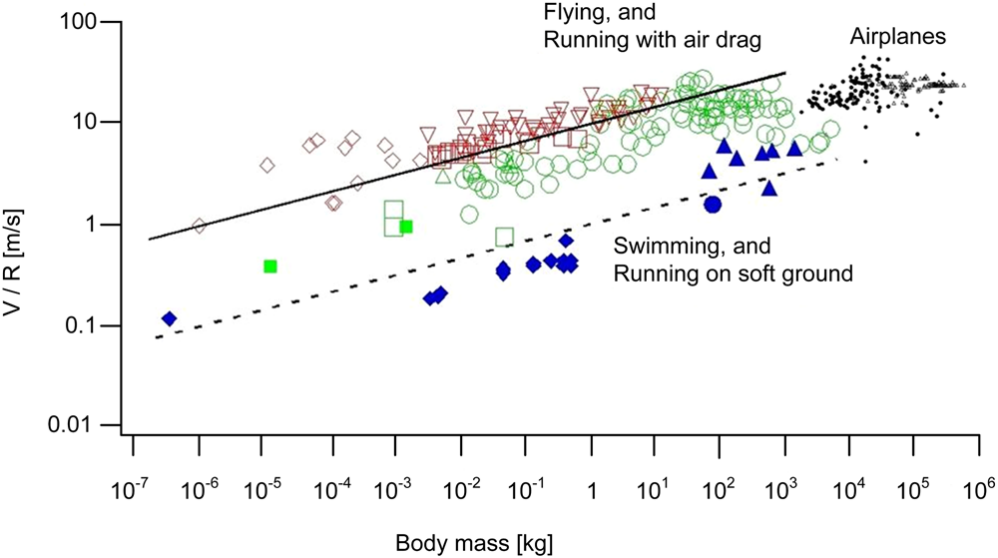
Revised version of Fig. 1, showing that the aircraft data line up with the animal fliers when the effect of lower air density and drag at higher altitudes is taken into account.

Compare Fig. 5 (bottom) with Fig. 5 (top). The speed data collapse on the theoretical trend obeyed by animals when the altitude effect (R) is taken into account. The correctness of the theory is strengthened if we replot the classical animal speed data of Fig. 1 as V/R versus M, in place of V versus M. This new representation is provided in Fig. 8. It is now evident why in the original version of Fig. 1 the airplane data deviate above the theoretical trend. With the effect of altitude taken into account, the theory covers correctly all the speed data, from animals (Fig. 1) to commercial and military aircraft (Figs 5–7). Noteworthy is also that the airplane data condensed now in the V/R-revised Fig. 5 (bottom) and Fig. 8 show a hump (i.e., a maximum speed at an intermediate size), which is reminiscent of the animal data plotted in Fig. 2.

## Why the fastest fliers are significantly smaller than the fastest swimmers

The animal data compiled in Fig. 2 show that the peak speeds of animals have the same order of magnitude (100 km/h), but the fastest flier is three orders of magnitude smaller than the fastest swimmer. Here is why this effect is in accord with the theory that concluded with Eqs (17–20):

Note that the body size for peak speed (M_peak_) is proportional to A raised to the power −1/(3/2–α), where α is positive and smaller than 1. In other words, the theoretical M_peak_ is expected to vary roughly as 1/A, where A is proportional to ${{\rm{C}}}_{2}^{3}$, or r^3^g^3/2^ρ^−1/2^. In conclusion, M_peak_ should vary roughly as r^−3^, that is in proportion with the density of the ambient, ρ_a_.

The density of the ambient decreases by a factor of 10^3^ from animal swimmers to animal fliers. Figure 2 shows that the body size of the fastest animals decreases by the same factor from swimmers to fliers. This suggests that the clouds of data for swimmers and fliers would fill the same cloud if on the abscissa the body sizes (M) are multiplied by r^3^, with r ~ 10 for fliers, r ~ 1 for swimmers, and an in-between r value such as 3 for runners.

The theory advanced in this paper is less conclusive with regard to the effect of the ambient on the peak speeds of animals, fliers versus swimmers. According to Eq. (18), the peak speeds should vary as ${{\rm{M}}}_{{\rm{peak}}}^{1/6}$, which means that V_peak_ should vary roughly as r^−1/2^, or (ρ/ρ_a_)^−1/6^. Taking r ≈ 10 for fliers and r ≈ 1 for swimmers, the prediction is that V_peak_ for fliers should be 1/3 of the V_peak_ for swimmers. The opposite trend is exhibited by the data in Fig. 2.

## Economies of scale

Bigger movers are more efficient movers. This aspect of the physics of powered locomotion is the basis of the present theory, starting with Eq. (2) with α < 1, and Fig. 3. This phenomenon is recognized more generally as economies of scale. Here we have the opportunity to show that the phenomenon is visible not only after plotting engine efficiencies versus size, as in Fig. 3, but also by looking at how airplanes are configured.

**Figure 9:**
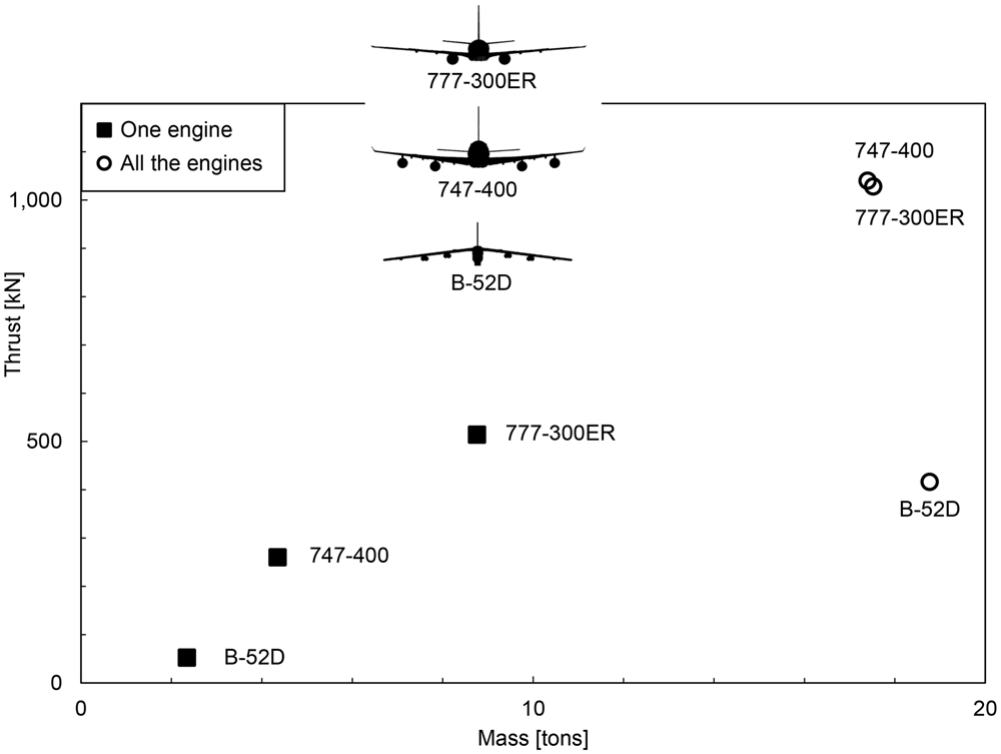
The evolution of the thrust and number of jet engines on three of the largest aircraft in service today (Table 1).

In Fig. 9 and Table 1 we used three airplane models to illustrate the evolution of the biggest transport aircraft that flies today. From the B52 to the B747 and the B777, the engine thrust (the force) and size (mass) increased by a factor of order 10. The number of engines decreased by a similar factor, from 8 to 4, and finally 2.

**Table 1:** Engine evolution in the three of the largest aircraft flying today^14–19^.

	B52-D	B747-400	B777-300ER
Year	1956	1984	2004
Aircraft range [km]	14,080	13,490	13,650
Aircraft fuel capacity [liters]	181,610	216,840	181,283
Aircraft loaded mass [kg]	120,000	396,890	351,533
Aircraft fuel efficiency [kg·km/l]	9,303	24,694	26,469
Engine	Pratt & Whitney J57-P-23	GE CFM-80C2	GE 90 115-B
Number of engines	8	4	2
Engine length [m]	6.19	4.27	7.28
Engine fan diameter [m]	0.99	2.69	3.3
Engine dry mass [kg]	2347	4350	8762
Engine thrust [kN]	52	260	513.9
Engine specific fuel consumption mg/(N·s)	59	9.32	8.31
Engine fuel heating value [MJ/kg]	43.15	44.65	44.65

From the point of view of the whole vehicle, the evolution means something different. The data on the right side of Fig. 9 show the total thrust and total mass of the engines that drive the airplane. The data line up on the vertical, which means that the B52 from 1956 is driven by the same engine mass as the B747 of 1984 and the B777 of 2004. The difference and the message are on the vertical. From the 1950s to the 1980s, the total engine mass is essentially the same, the total thrust increased by an order of magnitude, and the size of the vehicle increased by a factor of the same order, from the B52 to the B747 and B777.

In sum, the evolution has been toward bigger force, bigger size, and more efficient engines, over time.

## Concluding remarks

In this paper we relied on physics to show how the body size controls the maximum speed through the environment. The original challenge was to predict from theory that ‘outliers’ such as the cheetah must exist. The physical reason for outliers is that animals do not move the same way during their daily life. Grazing animals move constantly and eat constantly, while predators move and eat in spurts. In fact, predators spend most of their lives at rest, sleeping or watching the prey.

We showed that what accounts for the animal outlier (higher speed at smaller body mass) also accounts for the vehicle outlier. Military aircraft for chase, attack and reconnaissance are smaller and reach speeds higher than the biggest commercial aircraft. Yet, like the cheetah, the jet fighter spends most of its active life at rest, on the ground, out of sight.

The theoretical line pursued in this paper revealed two unexpected opportunities to complete the physics theory of animal and human flight. One was the deviation of the commercial aircraft data (upward, relative to animal data) in Fig. 1. The cause is the higher altitude of flying aircraft, where the air density is lower than close to the ground, and consequently the theoretical speeds must be higher. When the flying altitude effect is taken into account, the animal and aircraft data line up together (Fig. 8).

The second unexpected opportunity was to explain why the fastest animal fliers are 10^3^ times smaller than the fastest animal swimmers (Fig. 2). The explanation is in the theoretical formulas for maximum-speed locomotion. The body size for maximum speed depends on the density of the medium (ρ_a_) through which the animal is moving. The theoretical body size is approximately proportional to ρ_a_/ρ, and this ratio decreases by a factor of 10^3^ from the ρ_a_ of water to the ρ_a_ of air.

**Figure 10:**
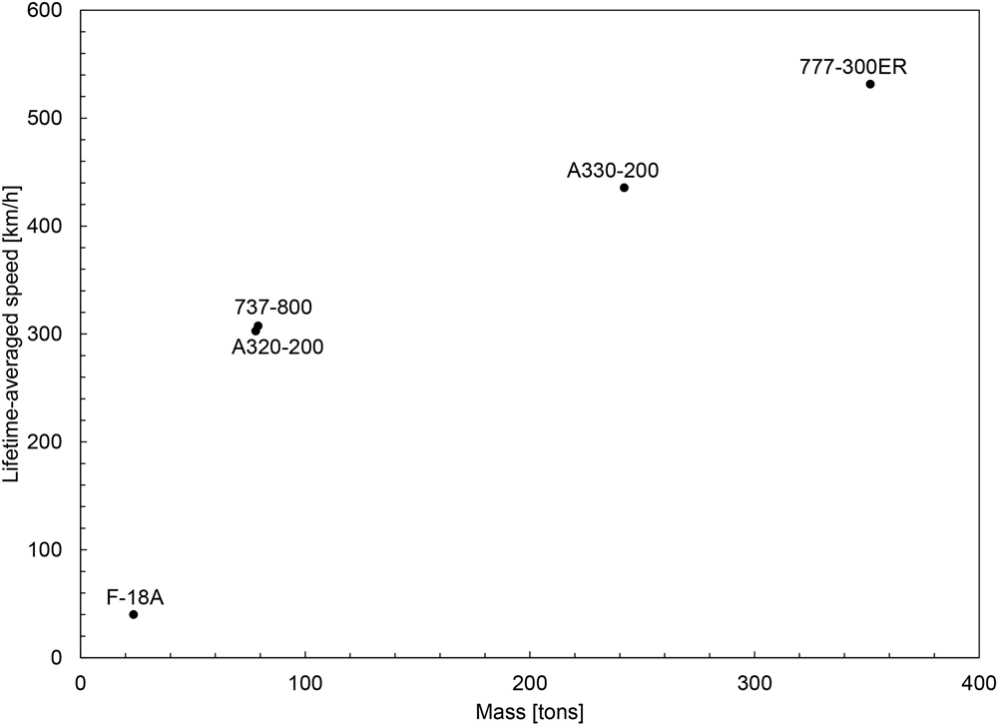
The lifetime-averaged speeds of current aircraft, showing the new ‘outlier’ position of the jet fighter (Table 2).

The practical usefulness of these theoretical advances is that they help us fast-forward the evolution of vehicle technology. This benefit was already demonstrated with the transition from the physics theory of all animal locomotion^1,4^, which revealed the main scaling laws of all moving animal design, to predicting the scaling laws of commercial airplanes^6^ and helicopters^7^, which have ‘converged’ naturally (from numerous disconnected designers and groups) during the documented lives of these technologies. It stands to reason that with the new theory and the morphological trends of modern aircraft reported in the present paper (e.g., sections 2–8 and Figs 5–10), designers, teams and industries will see more directly and more clearly the future of such vehicles. This predictive power of evolutionary design returns the favor to biology, for which it provides ample evidence of the natural phenomenon of evolution, which is observable in our lifetime^13^.

The speed ‘outlier’ phenomenon covered in this paper can be read from the much older perspective offered in Aesop’s fable “The Tortoise and the Hare”. What matters in the life of the mover is the movement (the territory covered, the speed averaged) over the lifetime. In Table 2 and Fig. 10 we brought together the lifetime speed and distance data of the work horses of commercial aviation and one jet fighter, the F-18A. This new presentation gives the word ‘outlier’ an entirely different meaning: the jet fighter is the outlier because during its lifetime it is slower than the bigger, the commercial aircraft. This new meaning is in fact the oldest, taught by Aesop.

**Table 2:** Lifetime aircraft speed, averaged over flight time plus ground time^14,20–27^.

	F-18A	737-800	A320-200	A330-300	B777-300ER
Loaded mass (kg)	23,541	79,016	78,000	242,000	351,533
Max speed (km/hr)	1,915	905	871	914	933
Cruise speed (km/hr)	1,062	842	829	871	892
Range (km)	2,017	5,436	6,100	11,750	13,650
Design flight service life (in air time only) (hrs)	10,000	60,000	60,000	100,000	125,000
Maximum hypothetical lifetime distance (10^6^ km)	10.6	50.5	49.7	87.1	115.0
Flight hours per year (hrs)	330	3,200	3,200	4,380	5,219.5
Expected total service life (in air time + ground time) (hrs)	265,454.5	164,250	164,250	200,000	209,790.21
Average speed over total aircraft life (km/hr)	40.01	307.58	302.83	435.5	531.48

## Electronic supplementary material

Supplementary Material
