# Supplementary material for: The fastest animals and vehicles are neither the biggest nor the fastest over lifetime
Source: Sci Rep. 2018 Aug 27;8:12925. doi: 10.1038/s41598-018-30303-1 (PMC6110785; doi:10.1038/s41598-018-30303-1)
Supplement: Supplementary file 1 — Supplementary Material [file 41598_2018_30303_MOESM1_ESM.docx]

**The fastest animals and vehicles are neither the biggest nor the fastest over lifetime**

**A. Bejan, Ü. Güneş, J. D. Charles and B. Sahin**

**Correspondence to** [**abejan@duke.edu**](mailto:abejan@duke.edu)

A. the data plotted in Fig. 3: Engine specifications of commercial airplanes [8].

| **Manufacturer** | **Model** | **Application(s)** | **Power (TO) [shp]** | **SFC (TO) [lb/shp hr]** | **Weight (dry) [lb]** | **Length [in]** | **Width/ Diameter [in]** |
| --- | --- | --- | --- | --- | --- | --- | --- |
| Allison | 250-C20B | Bell 47G, Bell 206B-1/B-3, AB206B, MD500D/E, A109A, BO105CBS/CBS-4/CBS-5, RH-1100, UH-12E, KA226 | 420 | 0.65 | 161 | 38.8 | 23.2 |
| Allison | 250-C20F | AS355E/F1/F2 | 420 | 0.65 | 161 | 38.8 | 23.2 |
| Allison | 250-C20J | Bell 206B-3/L | 420 | 0.65 | 161 | 38.8 | 23.2 |
| Allison | 250-C20R | Bell 206LT, Gemini ST, Bell 400/440 (not produced) | 450 | 0.608 | 173 | 38.8 | 23.2 |
| Allison | 250-C20R/1 | A109A Mk.2, A109C/C Max, Gemini ST | 450 | 0.608 | 173 | 38.8 | 23.2 |
| Allison | 250-C20R/2 | Bell 260B-3/L, MD500D/ER, MD520N, KA-226A | 450 | 0.608 | 173 | 38.8 | 23.2 |
| Allison | 250-C20R/3 | BO108 (not produced/see EC135) | 450 | 0.608 | 173 | 38.8 | 23.2 |
| Allison | 250-C20R/4 | Bell 206B-3/L | 450 | 0.608 | 173 | 38.8 | 23.2 |
| Allison | 250-C20R/9 |  | 450 | 0.608 | 173 | 38.8 | 23.2 |
| Allison | 250-B17C | Cessna 402/414, Nomad N-22/-24, BN-2T, P68TP, A36, GZ22, AT-35 | 420 | 0.657 | 198 | 44.9 | 22.5 |
| Allison | 250-B17D | SF260TP, KM2D, HTT34, CT-4C | 420 | 0.657 | 202 | 44.9 | 22.5 |
| Allison | 250-B17E | Nomad N-22/-24 | 420 | 0.657 | 202 | 44.9 | 22.5 |
| Allison | 250-B17F | AT-34, Grob 140TP | 450 | 0.61 | 212 | 44.9 | 22.5 |
| Allison | 250-C28B | Bell 206L-1, AB206L | 500 | 0.606 | 230 | 47.3 | 25.1 |
| Allison | 250-C28C | BO105LS/LS-A1/LS-A3 | 500 | 0.606 | 230 | 47.3 | 25.1 |
| Allison | 250-C30 | MD530, FT600, S-76A | 650 | 0.592 | 240 | 41 | 25.1 |
| Allison | 250-C30G | Bell 222 | 650 | 0.592 | 258 | 43.2 | 25.5 |
| Allison | 250-C30G/2 | Bell 230 | 650 | 0.592 | 258 | 43.2 | 25.5 |
| Allison | 250-C30M | AS350D | 650 | 0.592 | 258 | 43.2 | 25.5 |
| Allison | 250-C30P | Bell 206L-3/L-4 | 650 | 0.592 | 258 | 43.2 | 25.5 |
| Allison | 250-C30S | S-76A-2 | 650 | 0.592 | 258 | 43.2 | 25.5 |
| Allison | 250-C40B | Bell 430 | 715 | 0.57 | 280 | 41 | 25.1 |
| Allison | 501-D22A | Lockheed L-100-20/-30 | 4590 | 0.52 | 1834 | 146 | 44.6 |
| Allison (Rolls-Royce) | 250-C47B | Bell 407 | 650 | 0.58 | 274 | 41 | 25.1 |
| Allison (Rolls-Royce) | 250-C47M | MD600N | 650 | 0.58 | 274 | 41 | 25.1 |
| Allison (Rolls-Royce) | AE 2100A | Saab 2000 | 4152 | 0.46 | 1641 | 118 | 28.7 |
| Avco Lycoming | LTS 101-650C-2 | Bell 222 | 675 | 0.572 | 241 | 31.3 | 22.6 |
| Avco Lycoming | LTS 101-650C-3 | Bell 222 | 675 | 0.572 | 241 | 31.3 | 22.6 |
| Avco Lycoming | LTS 101-650C-3A | Bell 222 | 675 | 0.572 | 241 | 31.3 | 22.6 |
| Avco Lycoming | LTS 101-750C-1 | Bell 222B/UT | 735 | 0.577 | 244 | 31.3 | 22.6 |
| Avco Lycoming | LTS 101-600A-2 | AS350C/D/D1 | 615 | 0.571 | 253 | 30.9 | 22.4 |
| Avco Lycoming | LTS 101-600A-3 | AS350C/D/D1 | 615 | 0.582 | 265 | 31.5 | 22.4 |
| Avco Lycoming | LTS 101-750B-2 | AS366 | 742 | 0.57 | 268 | 32.4 | 24.7 |
| Avco Lycoming | LTS 101-750B-1 | BK117B/B1/B2 | 727 | 0.577 | 271 | 31.3 | 25.4 |
| Avco Lycoming | LTS 101-650B-1 | BK117A1/A3/A4 | 650 | 0.577 | 273 | 31.1 | 25.4 |
| Avco Lycoming | LTP 101-600A-1A | Cessna/Riley 421, Turbo Thrush, Ag-Cat, AT-302/-302A, Do 28D-5X | 615 | 0.544 | 325 | 37.4 | 21 |
| Avco Lycoming | LTP 101-700A-1A | Cessna/Riley 421, Turbo Thrush, Ag-Cat, Piaggio P166 | 700 | 0.544 | 335 | 37.4 | 21 |
| Avco Lycoming | T5311A | Bell 204B, AB204A | 1100 | 0.68 | 496 | 47.6 | 23 |
| Avco Lycoming | T5313B | Bell 204B-2, Bell 205A1 | 1400 | 0.58 | 544 | 47.6 | 23 |
| Avco Lycoming | T5317A | Bell 205A1, Kaman K-Max | 1500 | 0.59 | 564 | 47.6 | 23 |
| Avco Lycoming | LTC 4B-8D | Bell 214A | 2250 | 0.63 | 605 | 44 | 24 |
| Avco Lycoming | T5508D | Bell 214B/B-1/C | 2930 | 0.592 | 605 | 44 | 24 |
| Avco Lycoming (Textron Lycoming) | T55-L-714A | Boeing 414-100 Super D | 4867 | 0.5 | 832 | 48.5 | 28.7 |
| Baranov (OMSK) | TVD-20-03 | An-38-200, T-101V | 1380 | 0.506 | 628 | 69.7 | 33.5 |
| Garrett | TPE331-25D | PC-6/C Turbo-Porter | 575 | 0.665 | 335 | 46 | 26 |
| Garrett | TPE331-25/61 | MU-2A/2E1/25D, Heliporter 43, Hawk Commander 47, Volpar Super Turbo 18 | 575 | 0.665 | 335 | 46 | 26 |
| Garrett | TPE331-2-201C | C-212 | 715 | 0.556 | 336 | 43 | 26 |
| Garrett | TPE331-2-251A | Skyvan III | 715 | 0.556 | 336 | 43 | 26 |
| Garrett | TPE331-1-100F | Porter | 705 | 0.571 | 336 | 43 | 26 |
| Garrett | TPE331-1-101 | Interceptor 400, PC-6/C1 Turbo-Porter | 705 | 0.571 | 336 | 43 | 26 |
| Garrett | TPE331-1-101B | Turbo 18 (Turboliner) | 705 | 0.571 | 336 | 43 | 26 |
| Garrett | TPE331-1-101E | Jetliner 600 | 705 | 0.571 | 336 | 43 | 26 |
| Garrett | TPE331-1-101F | PC-6/C2-H2 Turbo-Porter, Peacemaker | 705 | 0.571 | 336 | 43 | 26 |
| Garrett | TPE331-1-101Z | S2R Turbo Thrush | 705 | 0.571 | 336 | 43 | 26 |
| Garrett | TPE331-1-151A | MU-2DP/F/G | 705 | 0.571 | 336 | 43 | 26 |
| Garrett | TPE331-1-151G | Merlin IIB | 705 | 0.571 | 336 | 43 | 26 |
| Garrett | TPE331-1-151K | Turbo Commander | 705 | 0.571 | 336 | 43 | 26 |
| Garrett | TPE331-1-151Z | G164B | 705 | 0.571 | 336 | 43 | 26 |
| Garrett | TPE331-3-301 |  | 840 | 0.548 | 355 | 43 | 26 |
| Garrett | TPE331-3-303 |  | 840 | 0.548 | 355 | 43 | 26 |
| Garrett | TPE331-3A-301W | Jetstream 3M (not produced) | 840 | 0.548 | 355 | 43 | 26 |
| Garrett | TPE331-6-251M | MU-2J/K/L/M | 808 | 0.577 | 355 | 43 | 26 |
| Garrett | TPE331-6-252B | King Air B100 | 808 | 0.577 | 355 | 43 | 26 |
| Garrett | TPE331-6-252L | Merlin IIB | 808 | 0.577 | 355 | 43 | 26 |
| Garrett | TPE331-6-252M | MU-2J/K/L/M, FU24-950/954, FU24A-950/954 | 808 | 0.577 | 355 | 43 | 26 |
| Garrett | TPE331-6-252T | Skyvan | 808 | 0.577 | 355 | 43 | 26 |
| Garrett | TPE331-6-253B | King Air B100 | 808 | 0.577 | 355 | 43 | 26 |
| Garrett | TPE331-6A-251M | MU-2J/K/L/M | 808 | 0.577 | 355 | 43 | 26 |
| Garrett | TPE331-6A-252M | MU-2J/K/L/M | 808 | 0.577 | 355 | 43 | 26 |
| Garrett | TPE331-5-251C | C-211-100 | 840 | 0.577 | 355 | 43 | 26 |
| Garrett | TPE331-5-251K | Turbo Commander 690/690A/680B | 840 | 0.577 | 355 | 43 | 26 |
| Garrett | TPE331-5-252C | C-212-100 | 840 | 0.577 | 355 | 43 | 26 |
| Garrett | TPE331-5-252D | Do 228-101/-201/-202/-202K | 840 | 0.577 | 355 | 43 | 26 |
| Garrett | TPE331-5-252K | Turbo Commander 690A/690B | 840 | 0.577 | 355 | 43 | 26 |
| Garrett | TPE331-5-252M | MU-2N/P | 840 | 0.577 | 355 | 43 | 26 |
| Garrett | TPE331-5-254K | Jetprop Commander 690C/690D | 840 | 0.577 | 355 | 43 | 26 |
| Garrett | TPE331-5-255K | Turbo Commander 690/690A/690B, Jetprop Commander 690C/690D | 840 | 0.577 | 355 | 43 | 26 |
| Garrett | TPE331-3U-303G | Merlin III/IIIA/IV/IVA, Metro | 904 | 0.548 | 355 | 43 | 26 |
| Garrett | TPE331-3U-303N | S55T | 904 | 0.548 | 355 | 43 | 26 |
| Garrett | TPE331-3U-303V | Jetstream 3 | 904 | 0.548 | 355 | 43 | 26 |
| Garrett | TPE331-3U-304G | Merlin III/IIIA/IV/IVA, Metro I/II/IIA | 904 | 0.548 | 355 | 43 | 26 |
| Garrett | TPE331-3UW-304G | Metro II/IIA | 904 | 0.548 | 355 | 43 | 26 |
| Garrett | TPE331-10A |  | 984 | 0.55 | 380 | 46 | 26 |
| Garrett | TPE331-10AV-511B | King Air B100 | 984 | 0.55 | 380 | 46 | 26 |
| Garrett | TPE331-10AV-511KA | Kilo Alpha 290 | 984 | 0.55 | 380 | 46 | 26 |
| Garrett | TPE331-10AV-511M | MU-2J/K/L/M, FU24-950/954, FU24A-950/954 | 984 | 0.55 | 380 | 46 | 26 |
| Garrett | TPE331-10G-511D | Do 228 | 984 | 0.55 | 380 | 46 | 26 |
| Garrett | TPE331-10GP-511D | Do 228 | 984 | 0.55 | 380 | 46 | 26 |
| Garrett | TPE331-10GT-511D | Do 228 | 984 | 0.55 | 380 | 46 | 26 |
| Garrett | TPE331-10N-511S | Conquest II | 984 | 0.55 | 380 | 46 | 26 |
| Garrett | TPE331-10N-512S |  | 984 | 0.55 | 380 | 46 | 26 |
| Garrett | TPE331-10N-513S |  | 984 | 0.55 | 380 | 46 | 26 |
| Garrett | TPE331-10N-514S |  | 984 | 0.55 | 380 | 46 | 26 |
| Garrett | TPE331-10N-515S |  | 984 | 0.55 | 380 | 46 | 26 |
| Garrett | TPE331-10N-531S | Conquest II | 984 | 0.55 | 380 | 46 | 26 |
| Garrett | TPE331-10N-532S |  | 984 | 0.55 | 380 | 46 | 26 |
| Garrett | TPE331-10N-533S |  | 984 | 0.55 | 380 | 46 | 26 |
| Garrett | TPE331-10N-534S |  | 984 | 0.55 | 380 | 46 | 26 |
| Garrett | TPE331-10N-535S |  | 984 | 0.55 | 380 | 46 | 26 |
| Garrett | TPE331-10P-511D | Do 228 | 984 | 0.55 | 380 | 46 | 26 |
| Garrett | TPE331-10R-501C |  | 984 | 0.55 | 380 | 46 | 26 |
| Garrett | TPE331-10R-502C |  | 984 | 0.55 | 380 | 46 | 26 |
| Garrett | TPE331-10R-511C | C-212-200 | 984 | 0.55 | 380 | 46 | 26 |
| Garrett | TPE331-10R-512C | C-212-200 | 984 | 0.55 | 380 | 46 | 26 |
| Garrett | TPE331-10R-513C | C-212-300 | 984 | 0.55 | 380 | 46 | 26 |
| Garrett | TPE331-10T-511D | Do 228-202K | 984 | 0.55 | 380 | 46 | 26 |
| Garrett | TPE331-10T-511K | Turbo Commander 690A/690B, Commander 840/900 | 984 | 0.55 | 380 | 46 | 26 |
| Garrett | TPE331-10T-511M | MU-2N/P | 984 | 0.55 | 380 | 46 | 26 |
| Garrett | TPE331-10T-512K |  | 984 | 0.55 | 380 | 46 | 26 |
| Garrett | TPE331-10T-513K |  | 984 | 0.55 | 380 | 46 | 26 |
| Garrett | TPE331-10T-515K |  | 984 | 0.55 | 380 | 46 | 26 |
| Garrett | TPE331-10T-516K | Turbo Commander 690A/690B, Commander 840/900 | 984 | 0.55 | 380 | 46 | 26 |
| Garrett | TPE331-10T-517K |  | 984 | 0.55 | 380 | 46 | 26 |
| Garrett | TPE331-10U-501G | Merlin IIIB | 984 | 0.55 | 380 | 46 | 26 |
| Garrett | TPE331-10U-502G |  | 984 | 0.55 | 380 | 46 | 26 |
| Garrett | TPE331-10U-503G | Merlin 300 (IIIC) | 984 | 0.55 | 380 | 46 | 26 |
| Garrett | TPE331-10U-511G | Merlin IIIB | 984 | 0.55 | 380 | 46 | 26 |
| Garrett | TPE331-10U-512G |  | 984 | 0.55 | 380 | 46 | 26 |
| Garrett | TPE331-10U-513G | Merlin 300 (IIIC) | 984 | 0.55 | 380 | 46 | 26 |
| Garrett | TPE331-10UA-511G | Metro II/IIA | 984 | 0.55 | 380 | 46 | 26 |
| Garrett | TPE331-10UA-511G | Metro II/IIA | 984 | 0.55 | 380 | 46 | 26 |
| Garrett | TPE331-10UF-501H |  | 984 | 0.55 | 380 | 46 | 26 |
| Garrett | TPE331-10UF-511H | Jetstream 3108 | 984 | 0.55 | 380 | 46 | 26 |
| Garrett | TPE331-10UF-512H |  | 984 | 0.55 | 380 | 46 | 26 |
| Garrett | TPE331-10UF-513H | Jetstream 3101/3103/3107 | 984 | 0.55 | 380 | 46 | 26 |
| Garrett | TPE331-10UF-514H |  | 984 | 0.55 | 380 | 46 | 26 |
| Garrett | TPE331-10UF-515H | Jetstream 3101 | 984 | 0.55 | 380 | 46 | 26 |
| Garrett | TPE331-10UF-516H |  | 984 | 0.55 | 380 | 46 | 26 |
| Garrett | TPE331-10UG-513H | Jetstream 3101/3102/3108 | 984 | 0.55 | 380 | 46 | 26 |
| Garrett | TPE331-10UG-514H | Jetstream 3101/3102 | 984 | 0.55 | 380 | 46 | 26 |
| Garrett | TPE331-10UG-515H |  | 984 | 0.55 | 380 | 46 | 26 |
| Garrett | TPE331-10UG-516H |  | 984 | 0.55 | 380 | 46 | 26 |
| Garrett | TPE331-10UGR-513H | Jetstream 3101/3102 | 984 | 0.55 | 380 | 46 | 26 |
| Garrett | TPE331-10UGR-514H | Jetstream 3101/3102/3109 | 984 | 0.55 | 380 | 46 | 26 |
| Garrett | TPE331-10UGR-515H | Jetstream 3112 | 984 | 0.55 | 380 | 46 | 26 |
| Garrett | TPE331-10UGR-516H | Jetstream 3112 | 984 | 0.55 | 380 | 46 | 26 |
| Garrett | TPE331-10UR-513H | Jetstream 3101/3102/3103/3112 | 984 | 0.55 | 380 | 46 | 26 |
| Garrett | TPE331-10UK |  | 1045 | 0.55 | 380 | 46 | 26 |
| Garrett | TPE331-11U-601G | Metro III, Metro 23-11 | 1000 | 0.53 | 400 | 46 | 26 |
| Garrett | TPE331-11U-602G |  | 1000 | 0.53 | 400 | 46 | 26 |
| Garrett | TPE331-11U-611G | Metro III, Metro 23-11 | 1000 | 0.53 | 400 | 46 | 26 |
| Garrett | TPE331-11U-612G | Metro III, Metro 23-11 | 1000 | 0.53 | 400 | 46 | 26 |
| Garrett | TPE331-12 |  | 1151 | 0.522 | 400 | 46 | 26 |
| Garrett | TPE331-12JR-701C | C-212-400 | 1151 | 0.522 | 400 | 46 | 26 |
| Garrett | TPE331-12JR-701S | Caravan | 1151 | 0.522 | 400 | 46 | 26 |
| Garrett | TPE331-12U-701G | Metro 23-12, Merlin 5/6 (not produced) | 1151 | 0.522 | 400 | 46 | 26 |
| Garrett | TPE331-12UA-701G | Metro 23-12 | 1151 | 0.522 | 400 | 46 | 26 |
| Garrett | TPE331-12UA-701H | Jetstream 3201/3201EP/3206 | 1151 | 0.522 | 400 | 46 | 26 |
| Garrett | TPE331-12UAR-701G | Metro V, Metro 23-12 | 1151 | 0.522 | 400 | 46 | 26 |
| Garrett | TPE331-12UH-701G | Metro 23-12 | 1151 | 0.522 | 400 | 46 | 26 |
| Garrett | TPE331-12UHR-701G | Metro 23-12 | 1151 | 0.522 | 400 | 46 | 26 |
| Garrett | TPE331-12UHR-701H | Jetstream 3201/3217 | 1151 | 0.522 | 400 | 46 | 26 |
| Garrett (Allied-Signal) | TPE331-14B | Cheyenne 400 | 1312 | 0.515 | 620 | 53 | 32 |
| Garrett (Allied-Signal) | TPE331-14GR-801H | Jetstream 4102 | 1723 | 0.51 | 620 | 53 | 32 |
| Garrett (Allied-Signal) | TPE331-14GR-802H |  | 1723 | 0.51 | 620 | 53 | 32 |
| Garrett (Allied-Signal) | TPE331-14GR-805H | Jetstream 4101 | 1723 | 0.51 | 620 | 53 | 32 |
| Garrett (Allied-Signal) | TPE331-14HR-801H | Jetstream 4102 | 1723 | 0.51 | 620 | 53 | 32 |
| Garrett (Allied-Signal) | TPE331-14HR-802H |  | 1723 | 0.51 | 620 | 53 | 32 |
| Garrett (Allied-Signal) | TPE331-14HR-805H | Jetstream 4101 | 1723 | 0.51 | 620 | 53 | 32 |
| GE | CT58-GE-110-1 | S-62B/C, HH-52A, V107/II | 1400 | 0.62 | 440 | 20.7 | 63.3 |
| GE | CT7-2A | Bell 214ST, EH101 prototypes | 1625 | 0.473 | 442 | 47 | 26 |
| GE | CT7-2D | S-70 | 1625 | 0.473 | 442 | 47 | 26 |
| GE | CT7-2D1 | S-70 | 1625 | 0.473 | 466 | 47 | 26 |
| GE | CT7-6 | EH101 Mk.300/Mk.500 | 2000 | 0.47 | 493 | 48.2 | 26 |
| GE | CT7-7A | CN-235 | 1700 | 0.474 | 783 | 96 | 29 |
| IHI | CT58-IHI-110-1 | KV107/II-2/II-7 | 1400 | 0.62 | 440 | 20.7 | 63.3 |
| Ivchenko | AI-20M | Il-18D/E, Il-38, An-32 | 3900 | 0.44 | 2293 | 121.9 | 46.5 |
| LHTEC | CTP800-4T | Ayres LM200 | 2700 | 0.47 | 1140 | 69 | 50 |
| Mitsubishi | CT63-M-5A | HS-369 | 317 | 0.65 | 139 | 41 | 19 |
| Pratt Whitney Canada | PT6A-11 | Cheyenne I/IA, T-1040 | 500 | 0.647 | 328 | 62 | 19 |
| Pratt Whitney Canada | PT6A-21 | King Air C90B | 550 | 0.63 | 328 | 62 | 19 |
| Pratt Whitney Canada | PT6A-27 | DHC-6 Twin Otter 300/320, Westwind II/III, Airliner A99, L-410A/F, EMB-110, Y-12 II, PC-6/B2 | 620 | 0.602 | 328 | 62 | 19 |
| Pratt Whitney Canada | PT6A-15AG | Turbo-Thrush S2R-T15, Turbo-Cat, Ag-Cat D, AT-400/-402/-502 | 680 | 0.602 | 328 | 62 | 19 |
| Pratt Whitney Canada | PT6A-28 | King Air E90/A100, Cheyenne II, EMB-121A Xingu I | 680 | 0.602 | 328 | 62 | 19 |
| Pratt Whitney Canada | PT6A-11AG | Turbo-Thrush S2R-T11, Ag-Cat, 620 TP | 500 | 0.647 | 330 | 62 | 19 |
| Pratt Whitney Canada | PT6A-36 | Commuter C99 | 750 | 0.59 | 331 | 62 | 19 |
| Pratt Whitney Canada | PT6A-34 | Commuter C99, 101/101B/102 Avara, ST-28, EMB-110K1/P1/P2 | 750 | 0.595 | 331 | 62 | 19 |
| Pratt Whitney Canada | PT6A-34AG | Ag-Cat, AT-402/502/503A, Fieldmaster, Turbo-Thrush S2R-T34 | 750 | 0.595 | 331 | 62 | 19 |
| Pratt Whitney Canada | PT6A-112 | Conquest I, Corsair I, F406 Caravan II | 500 | 0.637 | 334 | 62 | 19 |
| Pratt Whitney Canada | PT6A-25C | PC-7 Mk.II | 750 | 0.595 | 335 | 62 | 19 |
| Pratt Whitney Canada | PT6A-25A | PC-7, Firecracker | 550 | 0.63 | 343 | 62 | 19 |
| Pratt Whitney Canada | PT6A-135 | King Air F90, EMB-121A1 Xingu II, Cheyenne IIXL, Comanchero 750 | 750 | 0.585 | 344 | 62 | 19 |
| Pratt Whitney Canada | PT6A-135A | King Air F90-1, ST-50, Seastar | 750 | 0.585 | 344 | 62 | 19 |
| Pratt Whitney Canada | PT6A-114 | Caravan I/IA | 600 | 0.64 | 350 | 62 | 19 |
| Pratt Whitney Canada | PT6B-36 | S-76B | 981 | 0.594 | 372 | 59.2 | 19.5 |
| Pratt Whitney Canada | PT6A-41 | King Air 200, Cheyenne III | 850 | 0.591 | 403 | 67 | 19 |
| Pratt Whitney Canada | PT6A-42 | King Air B200, EMB-121V Xingu III | 850 | 0.601 | 403 | 67 | 19 |
| Pratt Whitney Canada | PT6A-61 | Cheyenne IIIA | 850 | 0.591 | 429 | 67 | 19 |
| Pratt Whitney Canada | PT6A-45A | Shorts 330, Mowhawk 298 | 850 | 0.554 | 434 | 72 | 19 |
| Pratt Whitney Canada | PT6A-45B | Shorts 330, Mowhawk 298 | 850 | 0.554 | 434 | 72 | 19 |
| Pratt Whitney Canada | PT6A-45R | Shorts 330-200/360-100, Mowhawk 298 | 850 | 0.553 | 448 | 72 | 19 |
| Pratt Whitney Canada | PT6A-62 | Orlik | 950 | 0.567 | 454 | 70 | 19 |
| Pratt Whitney Canada | PT6A-66 | Avanti, M-102 | 850 | 0.62 | 470 | 70 | 19 |
| Pratt Whitney Canada | PT6A-60A | King Air 300, King Air 350 | 1050 | 0.548 | 475 | 72 | 19 |
| Pratt Whitney Canada | PT6A-65B | Commuter 1900, PZL-M-18, Be-32 | 1100 | 0.536 | 481 | 74 | 19 |
| Pratt Whitney Canada | PT6A-65R | Shorts 360-200, AMI DC-3 | 1376 | 0.512 | 481 | 75 | 19 |
| Pratt Whitney Canada | PT6A-65AG | Turbo Thrush, AT-802/802A, Firemaster | 1300 | 0.509 | 486 | 75 | 19 |
| Pratt Whitney Canada | PT6A-65AR | Shorts 360-200, AMI DC-3 | 1424 | 0.509 | 486 | 75 | 19 |
| Pratt Whitney Canada | PT6A-67 | RC-12K/N/P/Q | 1100 | 0.547 | 506 | 76 | 19 |
| Pratt Whitney Canada | PT6A-67R | Shorts 360-300, Basler Turbo BT-67 | 1424 | 0.52 | 515 | 76 | 19 |
| Pratt Whitney Canada | PT6A-50 | DHC-7 Dash 7 1/100/101/150/151 | 1120 | 0.56 | 607 | 84 | 19 |
| Pratt Whitney Canada | PT6T-3B | Bell 212 | 1290 | 0.596 | 660 | 67 | 44 |
| Pratt Whitney Canada | PT6T-3B-1 | Bell 412/412SP | 1800 | 0.596 | 660 | 67 | 44 |
| Pratt Whitney Canada | PT6T-6 | S-58T, AB212 | 1875 | 0.602 | 660 | 67 | 44 |
| Pratt Whitney Canada | PT6T-3D | Bell 412EP | 1800 | 0.601 | 690 | 67 | 44 |
| Pratt Whitney Canada | PW118 | EMB-120/-120ER | 1800 | 0.498 | 861 | 81 | 31 |
| Pratt Whitney Canada | PW118A | EMB-120/-120ER | 1800 | 0.504 | 866 | 81 | 31 |
| Pratt Whitney Canada | PW119B | Do 328-100/-110 | 2180 | 0.49 | 916 | 81 | 31 |
| Pratt Whitney Canada | PW120 | ATR42, SA210TA | 2000 | 0.485 | 921 | 84 | 31 |
| Pratt Whitney Canada | PW120A | Dash 8-100/-100A | 2000 | 0.485 | 933 | 84 | 31 |
| Pratt Whitney Canada | PW121 | Dash 8-100/-100A/-100B, ATR42 | 2150 | 0.476 | 936 | 84 | 31 |
| Pratt Whitney Canada | PW123 | Dash 8-300 | 2380 | 0.47 | 992 | 84 | 33 |
| Pratt Whitney Canada | PW123AF | CL-215/415 | 2380 | 0.47 | 992 | 84 | 33 |
| Pratt Whitney Canada | PW123B | Dash 8-300 | 2500 | 0.463 | 992 | 84 | 33 |
| Pratt Whitney Canada | PW123C | Dash 8-200A/-Q300 | 2150 | 0.483 | 1060 | 84 | 33 |
| Pratt Whitney Canada | PW123D | Dash 8-200B/-Q300 | 2150 | 0.483 | 1060 | 84 | 33 |
| Pratt Whitney Canada | PW125B | Fokker 50-100 | 2380 | 0.463 | 1060 | 84 | 33 |
| Pratt Whitney Canada | PW124B | ATR72 | 2380 | 0.468 | 1060 | 84 | 33 |
| Pratt Whitney Canada | PW126A | BAe ATP | 2662 | 0.461 | 1060 | 84 | 33 |
| Pratt Whitney Canada | PW126 | BAe ATP | 2653 | 0.463 | 1060 | 84 | 33 |
| Pratt Whitney Canada | PW127B | Fokker 50-400 | 2750 | 0.459 | 1060 | 84 | 33 |
| Pratt Whitney Canada | PW127C | Y7-200A | 2750 | 0.459 | 1060 | 84 | 33 |
| Pratt Whitney Canada | PW127F | ATR72-500 | 2750 | 0.459 | 1060 | 84 | 33 |
| PZL Rzeszów | TWD-10W | W-3 Sokol | 888 | 0.6 | 310 | 73.8 | 30.1 |
| PZL Rzeszów | TWD-10B | An-28 | 1011 | 0.57 | 661 | 81.1 | 35.4 |
| Rolls-Royce | Gem 42-1 Mk.204 | Westland 30 Series 100 | 1135 | 0.65 | 404 | 43.2 | 23.5 |
| Rolls-Royce | Tyne RTy.12 Mk.515/10 | CL-44D/D-4/J | 5095 | 0.39 | 2219 | 109 | 43.2 |
| Turbomeca | Arriel 1A | SA365C | 641 | 0.573 | 265 | 42.9 | 22.4 |
| Turbomeca | Arriel 1A1 | SA365C1 | 641 | 0.573 | 265 | 42.9 | 22.4 |
| Turbomeca | Arriel 1A2 | SA365C2 | 641 | 0.573 | 265 | 42.9 | 22.4 |
| Turbomeca | Arriel 1B | AS350B/BA | 641 | 0.573 | 265 | 42.9 | 22.4 |
| Turbomeca | Astazou III |  | 591 | 0.643 | 324 | 56.3 | 18 |
| Turbomeca | Astazou XVIIIA | SA360C | 591 | 0.54 | 341 | 52.2 | 27.5 |
| Turbomeca | TM 333-2B | Indian ALH | 1001 | 0.529 | 345 | 41.1 | 28 |
| Turbomeca | Makila 1A | AS332C/L | 1757 | 0.496 | 535 | 54.9 | 20.3 |
| Turbomeca | Makila 1A1 | AS332C1/L1 | 1877 | 0.481 | 535 | 54.9 | 20.3 |
| Walter | M 601 D | L-410UVP | 724 | 0.654 | 426 | 65.3 | 25.6 |
| Walter | M 601 B | L-410MA/UVP | 691 | 0.656 | 426 | 65.9 | 25.6 |
| Walter | M 601 E | L-410UVP-E | 751 | 0.649 | 441 | 65.9 | 25.6 |
| Walter | M 601 T | PZL-130TM/TB | 751 | 0.649 | 445 | 65.9 | 25.6 |
| Walter | M 601 F | L-420, M-101, Ae-270 | 778 | 0.633 | 445 | 65.9 | 25.6 |
| Walter | M 602 | L-610 | 1824 | 0.559 | 1257 | 105.1 | 34.3 |

B. The data plotted in Figs. 5 and 6: Speed and mass data for military aircraft models [10].

| **Year** | **Country** | **Name** | **Max Speed (km/h)** | **Weight Max (kg)** |
| --- | --- | --- | --- | --- |
| 1936 | The Netherlands | Fokker DXXI / D21 | 460 | 2050 |
| 1937 | UK | Hawker Hurrican | 520 | 3000 |
| 1937 | UK | Wellington | 410 | 11703 |
| 1938 | Romania | IAR 80 | 560 | 2980 |
| 1938 | The Netherlands | Fokker G.IA. | 475 | 4800 |
| 1938 | Germany | JU87B-1 Stuka | 340 | 4254 |
| 1939 | Germany | BF 109E-3 | 560 | 2665 |
| 1940 | Japan | A6M-5 Zero | 557 | 2742 |
| 1940 | USA | F4F Wildcat | 512 | 3607 |
| 1941 | USA | P-40 WarHawk | 555 | 3655 |
| 1941 | USSR | MiG-3 | 640 | 3350 |
| 1942 | USA | B-24 Liberator | 488 | 27216 |
| 1942 | USSR | Yak-9D | 602 | 3120 |
| 1943 | USA | B-17G Flying Fortress | 462 | 29710 |
| 1943 | USA | B-25J Mitchell | 438 | 15876 |
| 1943 | USA | B-29A Superfortress | 575 | 56245 |
| 1943 | USA | P-38J Lightning | 666 | 9806 |
| 1943 | USA | P-47D Thunderbolt | 687 | 7900 |
| 1943 | Italy | Macchi M.C. 205 Veltro | 650 | 3408 |
| 1943 | USA | F4U-1A Corsair | 671 | 6350 |
| 1943 | UK | Mosquito FB Mk-VI | 611 | 10115 |
| 1944 | UK | Spitfire Mk XIV | 721 | 3856 |
| 1944 | USA | P-51D Mustang | 703 | 5493 |
| 1944 | USA | F6F-5 Hellcat | 611 | 6991 |
| 1944 | Germany | Messerschmitt Me262 | 870 | 6400 |
| 1944 | Germany | FW 190D | 685 | 4840 |
| 1944 | Germany | Me-163 Komet | 960 | 4110 |
| 1945 | USA | F-8F Bearcat | 678 | 5873 |
| 1945 | USA | P-80 Shooting Star | 967 | 7646 |
| 1946 | UK | Vampire FB Mk.6 | 882 | 5620 |
| 1947 | USA | A-1H Skyraider | 518 | 11340 |
| 1948 | USSR | MiG-15 Fagot | 1075 | 6045 |
| 1948 | USA | F-86A Sabre | 1089 | 7359 |
| 1949 | UK | Gloster Meteor F Mk.8 | 962 | 7122 |
| 1951 | Sweden | J29 Tunnan | 1035 | 7530 |
| 1952 | USA | F-84F Thunderstreak | 1060 | 12247 |
| 1953 | UK | Hawker Hunter FGA.9 | 1144 | 11158 |
| 1954 | USSR | MiG-17F Fresco-C | 1145 | 6075 |
| 1955 | USA | B-52G Stratofortress | 957 | 221253 |
| 1955 | USSR | MiG-19 Farmer | 1450 | 9100 |
| 1956 | France | Super Mystere B2 | 1200 | 10000 |
| 1956 | USA | F-100C Super Sabre | 1350 | 15100 |
| 1956 | USA | F-102 Delta Dagger | 1328 | 14288 |
| 1957 | USA | F-101 VooDoo | 1965 | 23768 |
| 1957 | Italy | G.91 | 1090 | 5670 |
| 1958 | Sweden | J32B Lansen | 988 | 13500 |
| 1958 | Canada | CF-105 Avro Arrow | 2104 | 3118 |
| 1958 | USA | F-8E Crusader | 1976 | 13154 |
| 1958 | USA | F-104G Starfighter | 2334 | 13170 |
| 1958 | USA | F-105D Thunderchief | 2204 | 23967 |
| 1959 | USA | B-58 Hustler | 2229 | 73936 |
| 1959 | USSR | MiG-21F Fishbed-C | 2175 | 8625 |
| 1959 | USA | F-106 Delta Dart | 2020 | 18974 |
| 1960 | UK | BAC Lightning F.6 | 2271 | 18900 |
| 1960 | UK | Vulcan B Mk.2 | 1030 | 113400 |
| 1963 | USA | A-6E Intruder | 1043 | 27400 |
| 1964 | France | Mirage III-E | 2350 | 13700 |
| 1964 | USA | A-5 Vigilante | 2572 | 29937 |
| 1964 | USA | XB-70 Valkyrie | 3200 | 249470 |
| 1965 | Sweden | J35 Draken | 2450 | 16000 |
| 1965 | UK | Buccaneer | 1127 | 28123 |
| 1966 | USA | SR-71 Blackbird | 3710 | 77110 |
| 1966 | USA | A-4 SkyHawk | 1100 | 11113 |
| 1967 | USA | U2-R | 980 | 18733 |
| 1967 | USA | F-4E Phantom II | 2410 | 17964 |
| 1968 | USA | A-7D Corsair II | 1112 | 19050 |
| 1969 | UK | Hawker Harrier GR3 | 1186 | 11340 |
| 1969 | USSR | SU17/SU22 Fitter | 2150 | 16950 |
| 1970 | France | Mirage F-1C | 2740 | 16200 |
| 1970 | USSR | MiG-25RB Foxbat-B | 3000 | 41200 |
| 1971 | Czechoslovakia | L-39C Albatros | 754 | 4700 |
| 1972 | USA | F-14A Tomcat | 2580 | 33724 |
| 1973 | USA | F-15 Eagle | 3062 | 30480 |
| 1973 | USA | F-5E Tiger II | 2009 | 11215 |
| 1973 | USSR | Mig-27M Flogger | 1700 | 20100 |
| 1973 | UK/France | Jaguar GR.1 | 2009 | 15700 |
| 1974 | USSR | SU24-MK Fencer | 2670 | 39700 |
| 1975 | Israel | F-21 Kfir C7 | 2445 | 16510 |
| 1976 | USA | A-10 Thunderbolt | 805 | 22680 |
| 1976 | USA | F-16 Falcon | 2511 | 17010 |
| 1976 | UK | BAe Hawk | 1000 | 7750 |
| 1977 | Sweden | JA-37 Viggen | 2124 | 17000 |
| 1978 | France | Super Etendard | 1347 | 11500 |
| 1978 | Europe | Alpha Jet | 1053 | 8000 |
| 1978 | USSR | SU-25 Frogfoot | 975 | 17600 |
| 1979 | France | Mirage 4000 | 2445 | 13000 |
| 1980 | USA | F/A-18 Hornet | 1900 | 22317 |
| 1980 | Europe | Panavia Tornado | 2333 | 27220 |
| 1981 | USA | EF-111A Raven | 2695 | 40370 |
| 1982 | USA | F-20 TigerShark | 2124 | 12475 |
| 1982 | USA | F-117 NightHawk | 1200 | 23814 |
| 1983 | USSR | MiG-31 Foxhound | 3000 | 46200 |
| 1983 | France | Mirage 2000C | 2878 | 17000 |
| 1984 | USSR | SU-27 Flanker | 2878 | 30000 |
| 1985 | USSR | MiG-29 Fulcrum | 2817 | 18000 |
| 1985 | USA | B-1B Lancer | 1375 | 216364 |
| 1987 | USSR | Tu-160 Blackjack | 2200 | 275000 |
| 1988 | Italy / Brazil | AMX | 1053 | 11500 |
| 1990 | USA | YF-23A Black Widow II | 1915 | 29029 |
| 1990 | USSR | Yak141 Freestyle | 1800 | 19500 |
| 1994 | Russia | SU-35 Super Flanker | 2480 | 34000 |
| 1994 | Taiwan | Ching-Kuo Indigenous Defense Fighter | 1275 | 12247 |
| 1996 | Russia | SU-37 Terminator | 2440 | 34000 |
| 1997 | USA | B-2 Spirit | 1103 | 181437 |
| 1997 | France | Rafale C | 2450 | 19500 |
| 1998 | Sweden | JAS-39 Gripen | 2205 | 12474 |
| 1999 | USA | F/A-18E Super Hornet | 2205 | 29937 |
| 2003 | Europe | EuroFighter Typhoon | 2450 | 23500 |
| 2005 | USA | F/A-22 Raptor | 2327 | 36228 |
| 2005 | China | Chengdu J-10 | 2450 | 18400 |
| 2006 | USA | V-22 Osprey | 510 | 27442 |
| 2007 | Russia | MiG-35 Fulcrum-F | 2756 | 29700 |
| 2007 | China/Pakistan | JF-17 Thunder, FC-1 Fierce Dragon | 1960 | 12700 |
| 2007 | India | Tejas | 2021 | 13500 |
